# Attitudes and Behaviors Toward Cardiopulmonary Resuscitation Among Healthcare and Non-healthcare Workers in Japan: A Cross-Sectional Study Using a Web-Based Questionnaire

**DOI:** 10.7759/cureus.76396

**Published:** 2024-12-25

**Authors:** Shinya Matsumoto, Yukio Tsugihashi, Takemi Akahane, Kiwamu Nagoshi, Manabu Akahane

**Affiliations:** 1 Department of Environmental Medicine and Public Health, Faculty of Medicine, Shimane University, Izumo, JPN; 2 Department of Public Health and Health Management and Policy, Nara Medical University, Kashihara, JPN; 3 Department of Gastroenterology, Nara Medical University, Kashihara, JPN; 4 Department of Health and Welfare Services, National Institute of Public Health, Wako, JPN

**Keywords:** cardiopulmonary resuscitation, occupation, out-of-hospital, web survey, willingness

## Abstract

Background

Cardiopulmonary arrest is a leading cause of death and requires swift intervention for survival. Previous studies have highlighted the critical importance of initiating cardiopulmonary resuscitation (CPR) and defibrillation within a limited timeframe. Improving outcomes depends on widespread CPR training, accessible automated external defibrillators (AEDs), and increased public awareness. Japan's universal healthcare system and accessible emergency medical services create an ideal environment for timely intervention. While global research has examined CPR hesitancy, few studies have focused on this issue in Japan. This study investigated willingness and attitudes toward CPR among various occupational groups in Japan, emphasizing the initial steps in responding to cardiopulmonary arrest.

Objective

This study explored the willingness and attitudes toward CPR among diverse occupational groups in Japan, focusing on the initial steps in responding to patients with cardiopulmonary arrest.

Methods

A cross-sectional survey was conducted through an Internet panel research company. Participants, stratified by occupation, age, and sex, completed a web-based questionnaire on CPR knowledge and willingness to act in a cardiac arrest scenario. Data were analyzed using univariate and multivariate linear regressions to identify factors influencing CPR attitudes and behaviors.

Results

Data from 1,648 respondents revealed differences in CPR attitudes and behaviors based on sex and occupational group. High resuscitation skills increased the likelihood of action, whereas concerns and worries reduced it. Self-evaluation of skills had a less significant effect. Variations existed in performing artificial respiration, with concerns about specific techniques influencing decisions. Multivariate regression analysis demonstrated an inverse relationship between the likelihood of action and worries about resuscitation. Sex and moral values also affected responses. Male medical doctors and nurses with lower moral values and higher resuscitation concerns were less likely to take action. However, they were more inclined to assist if the patient was familiar rather than unfamiliar.

Conclusions

The study identified notable differences in willingness and attitudes toward CPR between medical professionals (e.g., doctors and nurses) and non-medical professionals (e.g., the general public and care workers) as well as between men and women. Non-medical professionals were more likely to seek help when concerned about resuscitation techniques, whereas medical professionals showed hesitation due to worries about their performance. Women with uncertainties about resuscitation procedures were less likely to assist despite demonstrating a strong moral sense.

## Introduction

Cardiopulmonary arrest (CPA) is one of the most common causes of death in middle and old age, with a high mortality rate even when patients receive appropriate treatment, including immediate cardiopulmonary resuscitation (CPR), defibrillation such as automated external defibrillation, and emergency medical services (EMS) [1-4]. The interval from patient collapse to defibrillation is recognized as a critical survival factor, significantly influencing favorable neurological outcomes in CPA patients [3,5-9].

Weaver et al. [5] analyzed interview data that included the interval from patient collapse to CPR initiation and variables such as age, medical history, and drug use history. They emphasized the importance of minimizing the time to CPR initiation and the first countershock. Similarly, Valenzuela et al. [6] studied cases involving automated external defibrillation in US casinos and demonstrated that initiating defibrillation within three minutes significantly improved survival rates. Studies using nationwide data on out-of-hospital CPA in Japan have also shown that shorter intervals between collapse and CPR initiation are associated with better outcomes [7-11]. Both the speed and quality of CPR for patients with out-of-hospital CPA are critical factors in improving outcomes.

Improving outcomes for out-of-hospital CPA patients requires widespread participation in resuscitation training, the acquisition of basic CPR skills, and interventions to enable early CPR initiation. The widespread availability of automated external defibrillators (AEDs) is also essential for prompt defibrillation. In Japan, AEDs have been installed in public places such as large commercial facilities, schools, stations, and airports, where crowds often gather [12]. After using public access AEDs on out-of-hospital CPA patients, immediate contact with EMS is crucial, along with specialized emergency care and transportation to a hospital.

In Japan, EMS are municipal services accessible to all citizens free of charge [13]. Additionally, the country's universal health insurance system ensures that financial barriers do not prevent citizens from accessing EMS during critical situations such as CPA. In this context, improving survival and societal reintegration rates for out-of-hospital CPA patients requires more individuals to take the first steps, such as checking the patient's condition and contacting EMS promptly.

However, several barriers to rapid EMS contact and CPR initiation have been reported [7-9]. Many studies have examined the willingness, attitudes, and fears of performing CPR among both lay bystanders and healthcare professionals. Surveys conducted in conjunction with basic life support courses for the general public have identified reasons for CPR hesitation, including concerns about infection, lack of capability, legal issues, causing harm, and general fear. For instance, Savastano and Vanni [14] conducted a questionnaire-based survey after a basic life support course and reported these common concerns. Mäkinen et al. [15] surveyed healthcare professionals in Finland and Sweden, revealing similar factors that contributed to CPR hesitation, such as lack of confidence, anxiety, and perceived risks of harm.

Despite this body of research, few studies have focused on the willingness, attitudes, and fears of performing CPR among both lay bystanders and medical professionals in Japan. Specifically, little is known about how the intention to take initial actions, such as checking a patient's condition and contacting EMS, relates to individual CPR skill levels and concerns about performing CPR. This study aimed to investigate factors influencing willingness and attitudes toward performing CPR, including occupation, knowledge, and personal beliefs.

## Materials and methods

Data acquisition

A cross-sectional survey was conducted using an Internet panel provided by Macromill Co., Ltd. (approximately 1.3 million registered users). Individuals registered with the company received an email invitation to participate in the survey. Registration closed once the target sample size of 103 was reached for each occupational group (general public (non-medical), medical doctors, nurses, and care workers) and each 10-year age increment. Participants completed the survey through a web-based questionnaire and received a small cash reward upon completion; the exact amount of the reward was kept confidential by the survey company. The survey was conducted between December 13 and 15, 2019.

Questionnaire items

A custom-designed questionnaire was developed in collaboration with an expert in EMS research. A preliminary pilot survey was conducted with 15 individuals (approximately 1% of the total sample size) to evaluate the questionnaire's completeness, clarity, and consistency. Based on the pilot test findings, adjustments were made to enhance its coherence. The final version of the questionnaire was implemented as web-based survey screens created by a dedicated survey company.

When participants initially registered with the panel research company, they provided demographic information, including their age and sex. For this survey, participants were asked about their level of knowledge regarding CPR, their willingness to take action if a CPA occurred in front of them, their concerns about performing CPR, and their self-evaluations. The questionnaire items listed below (Table 1) were answered using a 6-point scale and served as the basis for analysis.

**Table 1:** Survey questionnaire items. AED: automated external defibrillator; CPR: cardiopulmonary resuscitation

Q-ID	Question statement
Q1	Please answer about your own resuscitation skills (level)
Q1-S1	I can perform chest compressions (cardiac massage)
Q1-S2	I can perform artificial respiration (e.g., mouth-to-mouth)
Q1-S3	I can use an AED
Q1-S4	I can determine if CPR is necessary
Q1-S5	I can perform resuscitation techniques calmly without panic
Q2	If an unfamiliar person experiences cardiopulmonary arrest in front of you, what would you do?
Q2-S1	Call out to check the person's condition
Q2-S2	Call emergency services (119 as the emergency number)
Q2-S3	Perform chest compressions (cardiac massage)
Q2-S4	Perform artificial respiration (e.g., mouth-to-mouth) if necessary
Q2-S5	Use an AED if available nearby
Q3	If a family member or close acquaintance experiences cardiopulmonary arrest in front of you, what would you do?
Q3-S1	Call out to check the person's condition
Q3-S2	Call emergency services (119 as the emergency number)
Q3-S3	Perform chest compressions (cardiac massage)
Q3-S4	Perform artificial respiration (e.g., mouth-to-mouth) if necessary
Q3-S5	Use an AED if available nearby
Q4	When performing CPR, are you concerned about the following?
Q4-S1	Worry about being sued later
Q4-S2	Worry about experiencing psychological distress
Q4-S3	Worrying whether the resuscitation techniques are appropriate
Q4-S4	Worrying about being observed by others while performing resuscitation
Q4-S5	Worry about potential contamination when touching a bleeding person
Q4-S6	Worry about potential harassment issues
Q5	Please answer about yourself
Q5-S1	I am diligent
Q5-S2	I am sociable (outgoing)
Q5-S3	I have a strong sense of responsibility
Q5-S4	I have a strong sense of morality
Q5-S5	I am cooperative

Data analysis

**Figure 1:**
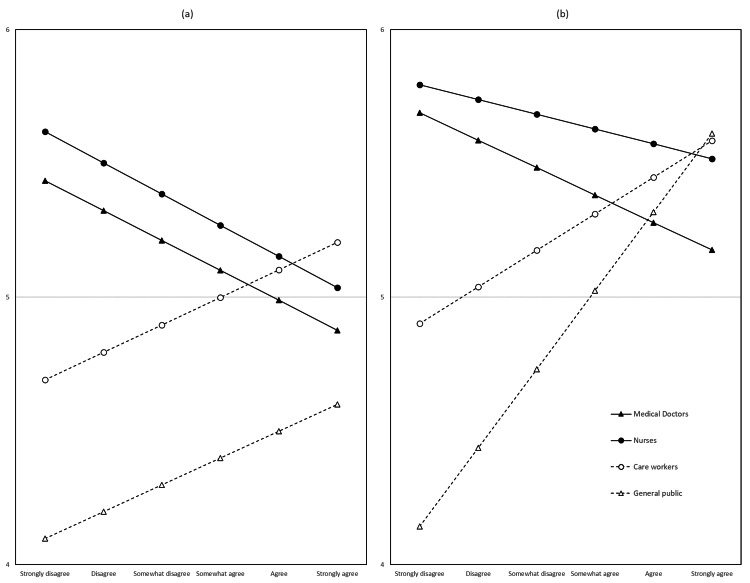
Results of substituting possible values of the explanatory variables into formulas derived from the multivariate linear regression analyses, shown with subfigures: (a) when the person experiencing cardiopulmonary arrest was unfamiliar and (b) when the person was familiar.

First, univariate linear regressions were performed by occupation, using Q2 and Q3 (Table 1) as the target variables. All items in Q2 and Q3 were treated as target variables, while items in Q1, Q4, and Q5 were used as explanatory variables. The t-statistics for the explanatory variables in all univariate linear regression results were selected and compared, as shown in Figure 1.

Second, two multivariate linear regressions were conducted with Q2S1 and Q3S1 as the target variables and Q4S3 as an explanatory variable, accounting for occupation. We assumed consistent variances across occupations and reduced the analysis's degrees of freedom. The target variable's values were determined by substituting the possible values of the explanatory variables into the established formula.

Third, a regression analysis was conducted using Q2S1 and Q3S1 as target variables, considering occupation and sex. Q4S3 and Q5S4 were treated as explanatory variables, and their relationships were examined by including their interaction terms. Similar to the second analysis, the target variable's values were calculated by substituting the possible values of the explanatory variables into the established formula.

Ethical considerations

This study was approved by the Ethics Committee of Nara Medical University (authorization number: 2238; approval date: June 25, 2019). An email outlining the survey details, including the survey period and genre, was sent en masse to the survey company's monitors. Monitors who expressed interest in participating were recruited and initially registered as potential participants. Registered participants received a detailed explanation of the survey's purpose, including the survey conductor (research institution and researchers' names), data storage methods and duration, and the method of publication of the analysis results (e.g., conference presentations and research papers). This information was provided via the Internet. Participants were then asked to provide their consent to participate, which served as informed consent. The final registration was completed through this process, and registration closed once the target number of participants for each cell by sex and age group was reached. Subsequently, the URL for the main survey was sent to the monitors who had provided informed consent.

## Results

A total of 1,648 respondents (412 from each occupational group) who completed all questions were included in the analysis. Table 2 presents the number of participants categorized by occupation and sex, highlighting that most medical doctors were male, while the majority of nurses were female. Additionally, Table 3 provides the number of participants categorized by occupation and age, showing an even distribution across age groups.

**Table 2:** Number of participants categorized by occupation and sex.

	Female	Male	Total
General public	242 (58.7%)	170 (41.3%)	412 (100%)
Medical doctor	113 (27.4%)	299 (72.6%)	412 (100%)
Nurse	359 (87.1%)	53 (12.9%)	412 (100%)
Care worker	255 (61.9%)	157 (38.1%)	412 (100%)
Total	969 (58.8%)	679 (41.2%)	1648 (100%)

**Table 3:** Number of participants categorized by occupation and age.

	25-29	30-34	35-39	40-44	45-49	50-54	55-59	60-64	Total
General public	47 (11.4%)	56 (13.6%)	52 (12.6%)	51 (12.4%)	64 (15.5%)	39 (9.5%)	48 (11.7%)	55 (13.3%)	412 (100%)
Medical doctors	34 (8.3%)	69 (16.7%)	46 (11.2%)	57 (13.8%)	51 (12.4%)	52 (12.6%)	53 (12.9%)	50 (12.1%)	412 (100%)
Nurses	40 (9.7%)	63 (15.3%)	47 (11.4%)	56 (13.6%)	74 (18%)	29 (7%)	72 (17.5%)	31 (7.5%)	412 (100%)
Care workers	40 (9.7%)	63 (15.3%)	56 (13.6%)	47 (11.4%)	60 (14.6%)	43 (10.4%)	65 (15.8%)	38 (9.2%)	412 (100%)
Total	161 (9.8%)	251 (15.2%)	201 (12.2%)	211 (12.8%)	249 (15.1%)	163 (9.9%)	238 (14.4%)	174 (10.6%)	1648 (100%)

Table 4 illustrates the t-statistics of each explanatory variable from the univariate linear regressions for each target variable. The explanatory variables are represented on a color scale, with red indicating positive values and blue indicating negative values, where darker shades correspond to larger absolute values. Notably, individuals with high resuscitation skills (Q1) were more likely to take action, as shown by the red blocks (positive t-statistics). Conversely, those who expressed worry (Q4) were less likely to take action, as indicated by blue blocks (negative t-statistics). While individuals with high self-evaluation (Q5) were also more likely to take action, the effect was less pronounced than the influence of resuscitation skills. Significant differences in the target variables across occupational groups were observed in Q2S4 and Q3S4 (performing artificial respiration, such as mouth-to-mouth resuscitation, if necessary). Additionally, explanatory variables with notable differences between occupational groups included Q4S3 (worrying about whether the resuscitation techniques were appropriate) and Q4S5 (worrying about being observed by others while performing resuscitation). The results of the multivariate linear regression analysis are shown in Figure 1. Among medical professionals (medical doctors and nurses) and non-medical professionals (the general public and care workers), the likelihood of taking action was inversely related to concerns about their resuscitation procedures. However, among the general public, greater concern about a technique was associated with a higher likelihood of taking action.

**Table 4:** T-statistics of the univariate linear regressions by target variables, explanatory variables, and occupations. AED: automated external defibrillator; CPR: cardiopulmonary resuscitation

				Explanatory variables	Q1					Q4						Q5				
					Please answer about your own resuscitation skills (level)					When performing CPR, are you concerned about the following?						Please answer about yourself				
					S1	S2	S3	S4	S5	S1	S2	S3	S4	S5	S6	S1	S2	S3	S4	S5
					I can perform chest compressions (cardiac massage)	I can perform artificial respiration (mouth-to-mouth, etc.)	I can use an AED	I can determine if CPR is necessary	I can perform resuscitation techniques calmly without panic	Worrying about being sued later	Worrying about experiencing psychological distress	Worrying if your resuscitation techniques are appropriate	Worrying about being observed by others while performing resuscitation	Worrying about potential contamination when touching a bleeding person	Worrying about potential harassment issues	I am diligent	I am sociable (outgoing)	I have a strong sense of responsibility	I have a strong sense of morality	I am cooperative
Target variables				Occupations																
Q2	When an unfamiliar person experiences cardiopulmonary arrest in front of you, what would you do?	S1	Call out to check the person's condition	General public	6.633	7.139	7.615	6.321	6.172	-1.058	-0.538	2.052	-2.197	1.552	-3.836	2.66	4.098	4.978	4.607	4.598
				Medical doctor	10.07	7.31	11.2	9.351	8.679	-1.93	-4.35	-3.128	-4.396	0.446	-2.573	4.498	2.84	5.725	5.56	5.212
				Nurse	9.462	6.134	9.054	8.624	6.255	-4.292	-4.164	-3.488	-6.855	0.702	-4.037	1.854	2.38	4.962	2.792	3.018
				Care worker	7.747	6.873	9.589	8.449	5.295	-0.411	-0.837	2.507	-2.601	2.103	-2.555	2.073	1.917	4.56	4.247	2.601
		S2	Call emergency services (119 as the emergency number)	General public	5.024	5.024	5.877	4.444	3.822	-1.127	-0.336	4.209	-0.021	2.676	-2.773	2.885	3.324	4.946	5.582	4.968
				Medical doctor	7.464	4.931	7.743	6.166	6.571	-2.782	-4.132	-3.649	-4.555	0.771	-2.83	4.243	1.589	5.15	4.609	3.159
				Nurse	8.574	5.121	9.313	7.557	5.483	-3.339	-3.093	-2.514	-5.069	0.34	-3.988	1.682	1.891	4.615	2.684	3.804
				Care worker	5.824	5.239	8.111	6.406	4.383	0.51	0.155	2.915	-1.849	2.98	-1.769	2.14	1.776	5.399	4.512	2.923
		S3	Perform chest compressions (cardiac massage)	General public	14.19	12.21	14.88	14.13	12.29	-2.582	-0.485	-2.478	-2.422	2.242	-0.63	3.807	3.367	3.154	2.46	4.094
				Medical doctor	14.044	9.179	13.605	12.23	11.68	-2.837	-4.287	-4.454	-4.052	0.498	-2.166	3.608	2.975	4.91	4.784	4.703
				Nurse	11.06	6.773	11.1	11.24	8.751	-4.876	-4.835	-5.002	-6.669	1.536	-4.183	1.538	2.801	4.146	2.473	3.31
				Care worker	15.41	12.14	14.29	13.5	10.9	-1.804	-2.466	-3.211	-4.76	0.112	-0.838	1.489	2.285	3.978	3.395	2.595
		S4	Perform artificial respiration (mouth-to-mouth, etc.) if necessary	General public	8.474	11.52	9.489	11.08	10.88	-1.753	-0.489	-3.572	-1.866	0.082	1.07	2.686	3.284	1.975	2.069	2.142
				Medical doctor	2.892	8.08	2.594	2.483	2.052	-0.587	-0.284	-0.215	-0.114	-4.765	-0.826	1.961	2.794	2.075	2.74	2.944
				Nurse	0.11	8.087	-0.563	1.232	2.505	-0.475	-0.181	-0.118	-0.909	-4.036	0.932	1.581	0.735	-0.257	2.109	0.789
				Care worker	8.697	13.595	8.274	8.625	8.21	-0.892	-0.945	-2.243	-2.969	-2.82	-0.403	2.064	2.724	1.937	3.465	1.706
		S5	Use an AED if available nearby	General public	10.83	9.505	14.1	10.54	10.41	-3.207	-1.656	-1.822	-3.336	2.132	-1.127	3.353	3.889	2.995	2.924	4.923
				Medical doctor	14.473	8.333	15.419	12.32	11.48	-2.472	-4.391	-4.326	-3.954	1.13	-2.424	3.773	1.568	3.931	3.896	3.262
				Nurse	10.59	6.947	15.24	11.5	8.179	-3.977	-3.494	-3.883	-5.757	2.55	-3.435	2.125	2.614	4.993	2.367	3.022
				Care worker	12.61	11.13	16.38	10.64	9.368	-2.566	-2.345	-1.68	-3.641	1.251	-1.398	1.928	3.438	4.974	4.729	3.112
Q3	When a family member or close acquaintance experiences cardiopulmonary arrest in front of you, what would you do?	S1	Call out to check the person's condition	General public	2.92	2.495	3.099	2.093	1.472	0.98	1.851	7.603	1.238	3.098	-1.679	1.736	2.261	3.958	4.213	4.088
				Medical doctor	13.62	8.262	12.73	10.79	9.251	-3.194	-4.854	-3.641	-4.988	3.202	-2.892	5.61	2.376	6.129	6.646	5.615
				Nurse	8.199	5.63	9.536	8.025	5.809	-2.985	-2.977	-2.043	-3.569	2.488	-3.26	2.001	1.573	5.201	3.603	3.228
				Care worker	6.232	5.02	8.216	5.515	3.909	-0.116	0.388	3.657	-1.235	3.224	-2.827	2.437	1.695	6.489	6.044	4.333
		S2	Call emergency services (119 as the emergency number)	General public	2.333	2.112	2.666	1.383	0.674	0.564	2.151	7.45	1.946	3.4	-1.842	0.553	1.838	3.672	3.951	4.691
				Medical doctor	13.1	7.918	11.35	10.08	8.844	-3.571	-5.042	-3.729	-4.841	3.168	-3.731	5.201	2.864	6.831	6.102	5.509
				Nurse	7.458	4.839	8.851	7.212	4.825	-2.27	-2.431	-1.385	-2.796	3.045	-3.264	2.417	1.774	4.66	3.603	3.041
				Care worker	5.219	4.621	7.387	4.809	3.125	-0.004	0.724	4.258	-0.859	3.476	-2.707	1.62	1.22	5.611	4.726	3.335
		S3	Perform chest compressions (cardiac massage)	General public	10.3	8.225	9.949	8.928	6.65	-0.227	0.58	0.769	-0.549	4.077	-1.286	2.43	4.334	3.362	2.832	4.88
				Medical doctor	15.83	9.279	15.91	13.63	12.31	-3.354	-4.513	-4.285	-5.114	2.731	-2.801	5.221	2.656	6.526	6.061	5.291
				Nurse	8.852	5.996	8.278	8.997	6.086	-3.891	-3.417	-2.386	-3.949	2.784	-4.372	2.155	2.775	5.042	4.425	3.959
				Care worker	11.84	9.105	13	10.21	7.742	-2.062	-2.469	-0.196	-4.166	1.733	-2.703	0.683	1.901	5.444	4.172	3.013
		S4	Perform artificial respiration (mouth-to-mouth, etc.) if necessary	General public	7.384	8.858	7.174	7.183	6.021	0.21	1.518	1.142	-0.088	3.413	-0.436	2.206	4.619	3.105	2.877	4.351
				Medical doctor	5.087	7.878	4.423	3.827	2.991	-1.289	-2.073	-0.794	-1.23	-2.197	-1.239	2.54	2.448	2.178	2.544	3.499
				Nurse	1.204	6.819	0.715	1.889	1.168	-0.94	-1.126	-0.128	-1.106	-0.983	-1.298	0.592	0.033	-0.346	1.676	0.168
				Care worker	8.186	10.55	8.424	6.459	5.784	-1.883	-1.226	-0.529	-3.23	-0.442	-1.737	1.635	2.671	5.332	4.405	3.021
		S5	Use an AED if available nearby	General public	7.811	5.895	10.05	7.113	6.323	0.089	1.214	2.129	0.135	4.779	-1.201	2.029	2.74	3.45	3.533	4.766
				Medical doctor	17.62	9.573	17.45	14.66	12.54	-2.792	-4.922	-4.23	-4.485	3.046	-2.434	4.568	1.622	5.039	5.178	4.631
				Nurse	8.1	5.059	10.58	8.897	6.123	-3.354	-3.819	-2.76	-3.626	2.65	-3.494	2.663	1.984	4.766	3.74	3.281
				Care worker	10.51	8.795	12.85	8.549	6.915	-2.35	-1.935	0.571	-3.389	1.38	-1.869	0.835	2.064	5.266	5.493	3.306

**Figure 2:**
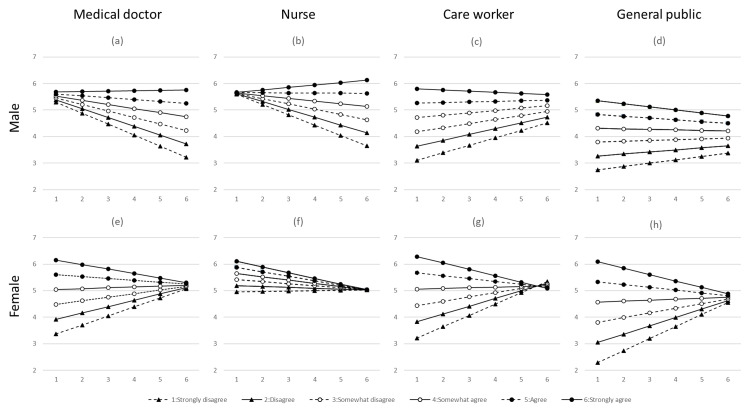
Results of substituting possible values of the explanatory variables into formulas derived from the multivariate linear regression analysis, where the target variable is "to an unfamiliar person," with subfigures segmented by both occupation and sex. The X-axis represents responses to "Q4S4: Worrying whether the resuscitation techniques are appropriate," and the Y-axis represents responses to "Call out to check the unfamiliar person's condition." The lines represent responses to "Q5S4: I have a strong sense of morality." Subfigures are as follows: (a) medical doctor, male; (b) nurse, male; (c) care worker, male; (d) general public, male; (e) medical doctor, female; (f) nurse, female; (g) care worker, female; and (h) general public, female.

Figure 2 shows the results obtained by substituting the possible values of the explanatory variables into the formulas derived from multivariate linear regression analyses, segmented by occupation and sex. The horizontal axis represents the responses to concerns about the appropriateness of the resuscitation techniques (Q4S3: Worrying whether the resuscitation techniques are appropriate), while the lines correspond to the responses indicating a strong sense of morality (Q5S4: I have a strong sense of morality). Notably, the line representing "people with strong moral values" was situated above the line for "people with weak moral values," indicating that people with strong moral values are more likely to take action. However, moral views did not affect women who scored high on Q4S3 (Q5S4). The results indicated that women with a strong sense of morality were less likely to take action if they were more anxious about the resuscitation procedure, as shown in the lower panel of Figure 2 (for women). The solid lines with black circles show a trend in which the Y-axis values decreased as the X-axis values increased. Among male medical doctors and nurses, those with lower Q4S3 scores were less affected by moral values. This suggests that male doctors and nurses tend to have difficulty communicating with people in distress if they are apprehensive about their resuscitation procedures. Furthermore, multivariate linear regression analysis was conducted with Q3S1 (call out to check the person's condition) as the target variable. The explanatory variables included Q4S3 (Worrying whether the resuscitation techniques are appropriate), Q5S4 (I have a strong sense of morality), and their interaction (the product of these two variables).

**Figure 3:**
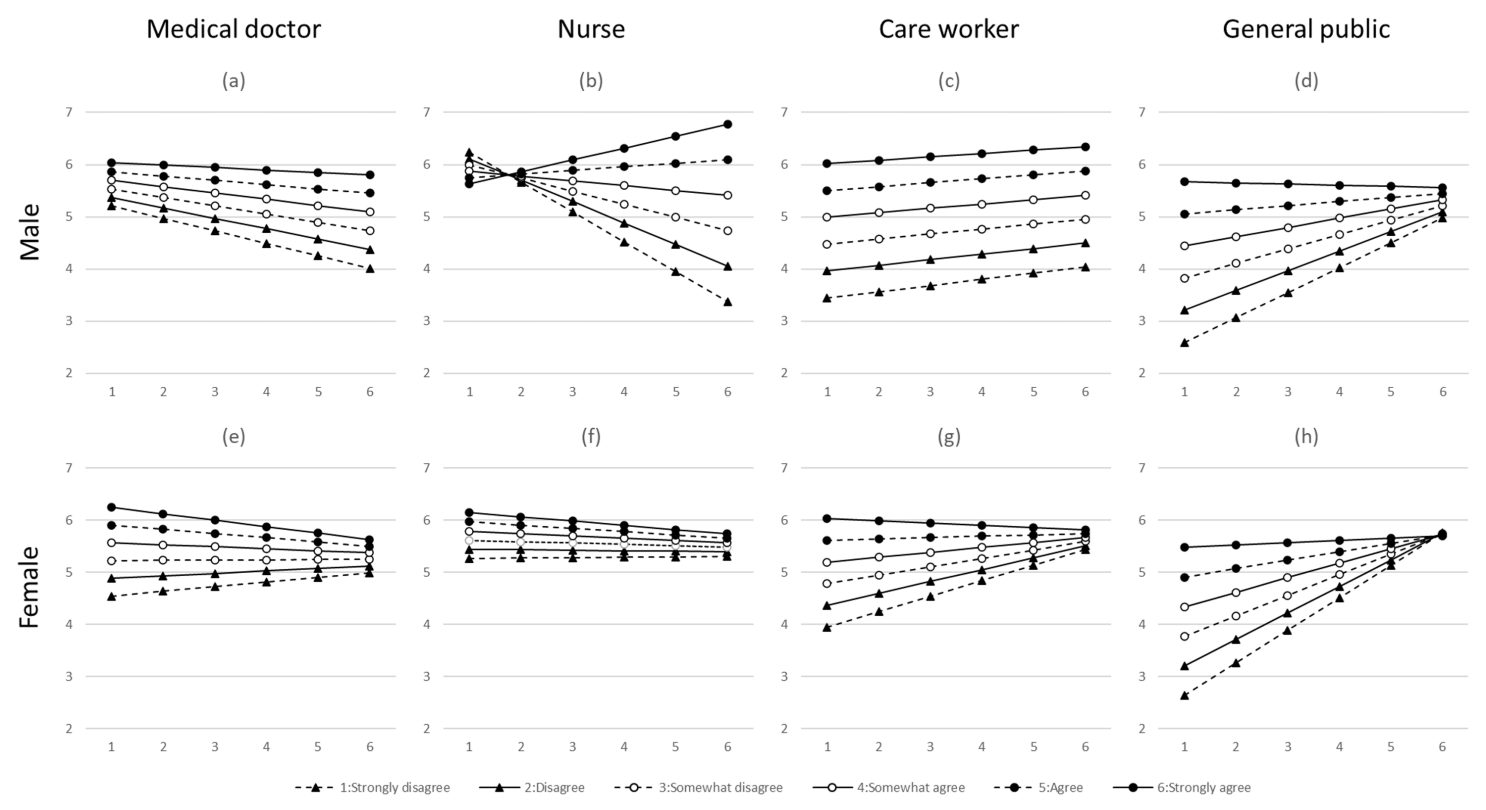
Results derived from the multivariate linear regression analysis, in which the target variable is "to a familiar person," with subfigures segmented by both occupation and sex. The X-axis represents the responses to "Q4S4: Worrying whether the resuscitation techniques are appropriate," and the Y-axis represents "Call out to check the familiar person's condition." The lines correspond to the answers to "Q5S4: I have a strong sense of morality." Subfigures are as follows: (a) medical doctor, male; (b) nurse, male; (c) care worker, male; (d) general public, male; (e) medical doctor, female; (f) nurse, female; (g) care worker, female; and (h) general public, female.

Figure 3 shows the values derived from the multivariate linear regression analysis. Male doctors and nurses who lacked a strong sense of morality and were concerned about the resuscitation procedure were less likely to call out to familiar persons, as shown in Figure 3. The dashed lines with black triangles in the two upper left graphs (male medical doctors and nurses) show the results, where the Y-axis values decrease as the X-axis values increase. However, they were still more likely to call out to a familiar person than to an unfamiliar person, as shown in Figure 3.

## Discussion

This study demonstrated significant differences between occupational groups and sexes in willingness and attitudes toward attempting life-saving treatments, such as CPR, for individuals needing help outside the hospital. The difference was particularly pronounced between medical professionals (medical doctors and nurses) and non-medical professionals (general public and care workers), especially in the adequacy of resuscitation techniques (Q4S3), in calling out to check the person's condition (Q2S1), in calling the EMS (Q2S2), and in the differences between men and women. Hesitation varied depending on whether the person encountering the CPA patient was a medical or non-medical professional and whether the person was a man or a woman. Hesitation in life-saving first aid measures can be a barrier to early treatment and access to EMS for patients with CPA and is significantly associated with improved survival and neurological outcomes. Therefore, the hesitation that impedes rapid CPR must be identified and addressed.

Regarding the differences between occupations, our results indicate that non-medical professionals tend to make emergency calls when concerned about the appropriateness of their resuscitation techniques. Moreover, non-medical workers were more likely to call for help when uncertain about the resuscitation procedure. Conversely, the likelihood of calling out was lower when there was no concern about the technique. In contrast, medical professionals were less likely to make an emergency call if they were worried about their resuscitation techniques. There was also a notable difference between occupations regarding concerns related to bleeding. Brenner and Kauffman [16] reported that medical workers hesitated to perform CPR using the mouth-to-mouth method because of the risk of infection. Boucek et al. [17] showed that healthcare workers were hesitant to use mouth-to-mouth breathing due to the fear of infectious diseases, such as human immunodeficiency virus (HIV). It is conceivable that differences in knowledge of infectious diseases may have contributed to these differences between medical and non-medical workers.

Furthermore, our results demonstrated that women were less likely to communicate with individuals in distress if they were uncertain about the resuscitation procedure, despite having a strong sense of morality. Daud et al. [18] reported that men were more motivated to perform CPR than women. However, there are few reports on whether there are differences in people's willingness to perform CPR based on their moral sense. This apparent contradiction between possessing a strong moral compass and hesitation to help someone in need raises important questions. For example, this hesitation might stem from positive intentions, such as considering whether someone else is better suited to help. Further research is required to explore this in more detail.

As shown in Figure 2, male physicians and nurses were more likely to take action if someone they knew was in distress, even if they were cautious and less morally inclined. When an acquaintance suffers from CPA, it can be difficult for medical doctors and nurses who do not have a strong moral sense, especially if they are uncertain about the CPR procedure. However, they were more willing to assist a familiar person than an unfamiliar one. Medical professionals are expected to initiate emergency calls and actively participate in resuscitation processes. This implies that they may be detained for an extended period. While a family member or close friend may not perceive a major problem in being detained for an extended period, they may hesitate about being detained by an unfamiliar individual. Although this is a potential concern, further investigation is required.

Many studies have reported attitudes and willingness to initiate CPR among laypersons and healthcare professionals [18-24]. Several studies have clarified the reasons why people hesitate to perform CPR for patients with CPA. Daud et al. [18] reported six factors, including socio-demographics (e.g., male sex, higher level of education, and married people), self-efficacy (e.g., confidence to perform CPR and to apply an AED), and legal obligations (e.g., legal liability protection), that were associated with willingness to perform CPR and use an AED. The most common barriers were fear of litigation and injury to the victims. Riccò et al. [22] reported that the reasons for unwillingness included inadequate knowledge and doubts about whether they could perform the techniques effectively. They concluded that knowledge of CPR techniques was the main predictor of the willingness to perform CPR. Jiang et al. [23] reported the willingness to perform CPR in different scenarios and the reasons for not performing compression-only or standard CPR. The willingness to perform compression-only and standard CPR was very high when the victim was a close relative, whereas the willingness of the general public to perform CPR on older adults was the lowest. The three primary reasons for not performing compression-only CPR were a lack of confidence, fear of harming the victim, and fear of causing legal problems. For standard CPR, the main obstacles were lack of confidence, which varied by sex, fear of disease transmission, and fear of harm to victims. Men mostly chose "fear of causing trouble (legal trouble)" as the major obstacle, whereas women mainly reported a lack of confidence. A few studies have reported that healthcare professionals might hesitate to perform CPR [15,24,25]. Huang et al. [24] reported that increased awareness of the importance of CPR may enhance the public's willingness to perform CPR on strangers, while healthcare personnel and women showed lower willingness to perform bystander CPR. They cited two possible reasons for the low motivation of healthcare workers. First, healthcare workers trained to perform artificial respiration and compression may feel obligated to perform artificial respiration on strangers, which could act as a strong disincentive for bystander CPR. Second, potential legal issues may have contributed to reluctance. This study also highlighted several factors that increased strangers' reported willingness to perform CPR, including the introduction of the limited "Good Samaritan immunity" for those who perform CPR and providing information such as the acceptability of chest compression-only CPR for sudden adult cardiac arrests and the understanding that delaying resuscitation may result in permanent brain injury.

Limitations

This study has several limitations. First, the data were collected only from respondents registered with an Internet panel survey company, which may have introduced sample bias. Respondents received a small amount of compensation and may have been influenced. Nonetheless, it is worth noting that panel surveys are increasingly used in research involving questionnaires [26-30]. Second, because this was a self-administered questionnaire, there may have been substantial variations in the results regarding individual personality traits, such as moral values. It is common for Japanese respondents to avoid extreme options on 6-point questionnaires. In the analysis, a diagram was created using the formula derived from multivariate linear regression to represent the relationship between the responses.

As a result, extreme combinations not present in the data may have been plotted. In other words, it represents an extrapolation of the data, which may pertain to rare cases. The respondents' personalities and actions in an emergency are interrelated, which may have influenced the survey results.

## Conclusions

This study focused on factors influencing the willingness and attitudes toward CPR among different occupational groups in Japan, including resuscitation skills, concerns, and moral values. We found that individuals with higher resuscitation skills and stronger moral values were more likely to take action, while those with concerns were less inclined. When comparing medical professionals to the general public, concerns about their resuscitation procedures were inversely related to taking action. Male medical doctors and nurses who lacked strong moral values and had concerns about resuscitation procedures tended to hesitate to assist individuals in distress.

## References

[1] Akahane M, Ogawa T, Tanabe S, Koike S, Horiguchi H, Yasunaga H, Imamura T (2012). Impact of telephone dispatcher assistance on the outcomes of pediatric out-of-hospital cardiac arrest. Crit Care Med.

[2] Naito H, Yumoto T, Yorifuji T (2021). Association between emergency medical service transport time and survival in patients with traumatic cardiac arrest: a nationwide retrospective observational study. BMC Emerg Med.

[3] Koike S, Ogawa T, Tanabe S (2011). Collapse-to-emergency medical service cardiopulmonary resuscitation interval and outcomes of out-of-hospital cardiopulmonary arrest: a nationwide observational study. Crit Care.

[4] Nishi T, Maeda T, Takase K, Kamikura T, Tanaka Y, Inaba H (2013). Does the number of rescuers affect the survival rate from out-of-hospital cardiac arrests? Two or more rescuers are not always better than one. Resuscitation.

[5] Weaver WD, Cobb LA, Hallstrom AP, Fahrenbruch C, Copass MK, Ray R (1986). Factors influencing survival after out-of-hospital cardiac arrest. J Am Coll Cardiol.

[6] Valenzuela TD, Roe DJ, Nichol G, Clark LL, Spaite DW, Hardman RG (2000). Outcomes of rapid defibrillation by security officers after cardiac arrest in casinos. N Engl J Med.

[7] Hara M, Hayashi K, Kitamura T (2017). Outcomes differ by first documented rhythm after witnessed out-of-hospital cardiac arrest in children: an observational study with prospective nationwide population-based cohort database in Japan. Eur Heart J Qual Care Clin Outcomes.

[8] Kiyohara K, Nishiyama C, Kitamura T (2019). The association between public access defibrillation and outcome in witnessed out-of-hospital cardiac arrest with shockable rhythm. Resuscitation.

[9] Shibahashi K, Ishida T, Kuwahara Y, Sugiyama K, Hamabe Y (2019). Effects of dispatcher-initiated telephone cardiopulmonary resuscitation after out-of-hospital cardiac arrest: a nationwide, population-based, cohort study. Resuscitation.

[10] Blewer AL, McGovern SK, Schmicker RH (2018). Gender disparities among adult recipients of bystander cardiopulmonary resuscitation in the public. Circ Cardiovasc Qual Outcomes.

[11] Blom MT, Oving I, Berdowski J, van Valkengoed IG, Bardai A, Tan HL (2019). Women have lower chances than men to be resuscitated and survive out-of-hospital cardiac arrest. Eur Heart J.

[12] Kobayashi D, Sado J, Kiyohara K (2020). Public location and survival from out-of-hospital cardiac arrest in the public-access defibrillation era in Japan. J Cardiol.

[13] Yanagawa Y, Sakamoto T (2010). Analysis of prehospital care for cardiac arrest in an urban setting in Japan. J Emerg Med.

[14] Savastano S, Vanni V (2011). Cardiopulmonary resuscitation in real life: the most frequent fears of lay rescuers. Resuscitation.

[15] Mäkinen M, Niemi-Murola L, Ponzer S, Kurola J, Aune S, Kurland L, Castrén M (2014). Healthcare professionals hesitate to perform CPR for fear of harming the patient. Resuscitation.

[16] Brenner BE, Kauffman J (1993). Reluctance of internists and medical nurses to perform mouth-to-mouth resuscitation. Arch Intern Med.

[17] Boucek CD, Phrampus P, Lutz J, Dongilli T, Bircher NG (2009). Willingness to perform mouth-to-mouth ventilation by health care providers: a survey. Resuscitation.

[18] Daud A, Nawi AM, Aizuddin AN, Yahya MF (2023). Factors and barriers on cardiopulmonary resuscitation and automated external defibrillator willingness to use among the community: a 2016-2021 systematic review and data synthesis. Glob Heart.

[19] Perman SM, Shelton SK, Knoepke C (2019). Public perceptions on why women receive less bystander cardiopulmonary resuscitation than men in out-of-hospital cardiac arrest. Circulation.

[20] Mejicano GC, Maki DG (1998). Infections acquired during cardiopulmonary resuscitation: estimating the risk and defining strategies for prevention. Ann Intern Med.

[21] Baubin M, Rabl W, Pfeiffer KP, Benzer A, Gilly H (1999). Chest injuries after active compression-decompression cardiopulmonary resuscitation (ACD-CPR) in cadavers. Resuscitation.

[22] Riccò M, Berrone M, Vezzosi L, Gualerzi G, Canal C, De Paolis G, Schallenberg G (2020). Factors influencing the willingness to perform bystander cardiopulmonary resuscitation on the workplace: a study from North-Eastern Italy. Acta Biomed.

[23] Jiang Y, Wu B, Long L, Li J, Jin X (2020). Attitudes and willingness toward out-of-hospital cardiopulmonary resuscitation: a questionnaire study among the public trained online in China. BMJ Open.

[24] Pei-Chuan Huang E, Chiang WC, Hsieh MJ (2019). Public knowledge, attitudes and willingness regarding bystander cardiopulmonary resuscitation: a nationwide survey in Taiwan. J Formos Med Assoc.

[25] Mäkinen M, Niemi-Murola L, Kaila M, Castrén M (2009). Nurses' attitudes towards resuscitation and national resuscitation guidelines--nurses hesitate to start CPR-D. Resuscitation.

[26] Matsumoto S, Kanagawa Y, Nagoshi K, Akahane T, Imamura T, Akahane M (2023). Consumer willingness to pay for food defense and food hygiene in Japan: cross-sectional study. Interact J Med Res.

[27] Sugiura H, Ohkusa Y, Akahane M, Sano T, Okabe N, Imamura T (2011). Development of a web-based survey for monitoring daily health and its application in an epidemiological survey. J Med Internet Res.

[28] Akahane M, Maeyashiki A, Tanaka Y, Imamura T (2019). The impact of musculoskeletal diseases on the presence of locomotive syndrome. Mod Rheumatol.

[29] Min YH, Lee JW, Shin YW (2014). Daily collection of self-reporting sleep disturbance data via a smartphone app in breast cancer patients receiving chemotherapy: a feasibility study. J Med Internet Res.

[30] Akahane M, Maeyashiki A, Yoshihara S, Tanaka Y, Imamura T (2016). Relationship between difficulties in daily activities and falling: Loco-Check as a self-assessment of fall risk. Interact J Med Res.

